# Sex Differences in Fractional Flow Reserve Utilization

**DOI:** 10.3390/jcm13144028

**Published:** 2024-07-10

**Authors:** Marta Bujak, Krzysztof Malinowski, Zbigniew Siudak, Anna Ćmiel, Maciej Lesiak, Stanisław Bartuś, Jacek Legutko, Wojciech Wańha, Adam Witkowski, Dariusz Dudek, Mariusz Gąsior, Robert Gil, Marcin Protasiewicz, Jacek Kubica, Piotr Godek, Wojciech Wojakowski, Paweł Gąsior

**Affiliations:** 1 Division of Cardiology and Structural Heart Diseases, Medical University of Silesia, 40-055 Katowice, Poland; cmiel.ania@gmail.com (A.Ć.); wojciechwanha@gmail.com (W.W.); piotrgodek77@gmail.com (P.G.); wojtek.wojakowski@gmail.com (W.W.); p.m.gasior@gmail.com (P.G.); 2 Department of Bioinformatics and Telemedicine, Faculty of Medicine, Jagiellonian University Medical College, 31-008 Krakow, Poland; krzysztof.piotr.malinowski@gmail.com; 3 Center for Digital Medicine and Robotics, Jagiellonian University Medical College, 31-008 Krakow, Poland; 4 Faculty of Medicine and Health Sciences, Jan Kochanowski University, 25-369 Kielce, Poland; zbigniew.siudak@gmail.com; 5 1st Department of Cardiology, Poznan University of Medical Sciences, 61-701 Poznan, Poland; mlesiak13@gmail.com; 6 2nd Department of Cardiology, Institute of Cardiology, Jagiellonian University Medical College, 31-008 Krakow, Poland; mbbartus@cyfronet.pl; 7 Department of Interventional Cardiology, Institute of Cardiology, Jagiellonian University Medical College, John Paul II Hospital, 31-202 Krakow, Poland; jacek.legutko@kcri.org; 8 Department of Interventional Cardiology and Angiology, National Institute of Cardiology, 04-628 Warszawa, Poland; witkowski@hbz.pl; 9 Institute of Cardiology, Jagiellonian University Medical College, 31-008 Krakow, Poland; mcdudek@cyfronet.pl; 10 3rd Department of Cardiology, School of Medicine with the Division of Dentistry in Zabrze, Medical University of Silesia, 40-055 Katowice, Poland; m.gasior@op.pl; 11 Silesian Center for Heart Diseases, 41-800 Zabrze, Poland; 12 Department of Cardiology, National Medical Institute of the Ministry of Interior and Administration, 02-507 Warsaw, Poland; scorpirg@gmail.com; 13 Institute of Heart Diseases, Wroclaw Medical University, 50-367 Wroclaw, Poland; marcin.protasiewicz@umw.edu.pl; 14 Department of Cardiology and Internal Medicine, Collegium Medicum, Nicolaus Copernicus University, 87-100 Toruń, Poland; jwkubica@gmail.com

**Keywords:** FFR, PCI, sex, physiological assessment

## Abstract

**Background**: The literature review shows that female patients are more frequently underdiagnosed or suffer from delayed diagnosis. Recognition of sex-related differences is crucial for implementing strategies to improve cardiovascular outcomes. We aimed to assess sex-related disparities in the frequency of fractional flow reserve (FFR)-guided procedures in patients who underwent angiography and/or percutaneous coronary intervention (PCI). **Methods**: We have derived the data from the national registry of percutaneous coronary interventions and retrospectively analyzed the data of more than 1.4 million angiography and/or PCI procedures [1,454,121 patients (62.54% men and 37.46% women)] between 2014 and 2022. The logistic regression analysis was conducted to explore whether female sex was associated with FFR utilization. **Results**: The FFR was performed in 61,305 (4.22%) patients and more frequently in men than women (4.15% vs. 3.45%, *p* < 0.001). FFR was more frequently assessed in females with acute coronary syndrome than males (27.75% vs. 26.08%, *p* < 0.001); however, women with chronic coronary syndrome had FFR performed less often than men (72.25% vs. 73.92%, *p* < 0.001). Females with FFR-guided procedures were older than men (69.07 (±8.87) vs. 65.45 (±9.38) *p* < 0.001); however. less often had a history of myocardial infarction (MI) (24.79% vs. 36.73%, *p* < 0.001), CABG (1.62% vs. 2.55%, *p* < 0.005) or PCI (36.6% vs. 24.79%, *p* < 0.001) compared to men. Crude comparison has shown that male sex was associated with a higher frequency of FFR assessment (OR = 1.2152–1.2361, *p* < 0.005). **Conclusions**: Despite a substantial rise in FFR utilization, adoption in women remains lower than in men. Female sex was found to be an independent negative predictor of FFR use.

## 1. Introduction

Female patients present with coronary artery disease (CAD) later in life, with different symptomatology and worse prognosis, compared to men. Despite advances in treatment and detection of CAD, and despite a significant drop in ischemic heart disease (IHD) mortality among women over the past few decades, sex-based disparities in outcomes persist, and CAD continues to be the leading cause of death in women [1].

The literature shows that female patients are more frequently underdiagnosed or receive delayed diagnosis in comparison to men. What is more, women more often receive suboptimal treatment and are underrepresented in clinical trials [2,3,4,5,6]. Recognition of sex-related differences is crucial for implementing strategies to improve cardiovascular outcomes in female patients. Adequate recognition of the full spectrum of IHD in women remains a barrier that has yet to be tackled by the medical community [7]. Physiological assessment using fractional flow reserve (FFR) is a key diagnostic tool to evaluate the functional severity of a lesion and guide intervention. It plays a crucial role in establishing the diagnosis and guiding further management. Multiple studies have confirmed its essential role in contemporary interventional cardiology, because the use of physiology assessment improves clinical outcomes. Current guidelines of the European Society of Cardiology (ESC) emphasize the role of the functional assessment of lesion severity in patients with intermediate stenosis. However, sex-specific recommendations have not yet been developed [8].

We sought to examine sex-related differences in clinical and procedural characteristics and the utilization of fractional flow reserve (FFR) in patients who underwent angiography and/or percutaneous coronary intervention (PCI).

## 2. Materials and Methods

**Figure 1:**
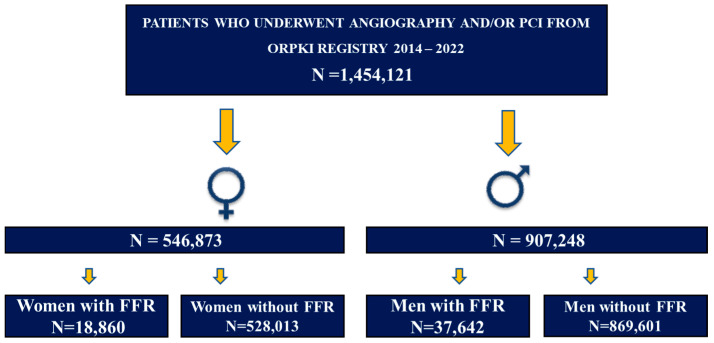
Study flowchart.

The current multicenter, retrospective analysis was performed using the data from the national registry of percutaneous coronary interventions (ORPKI), which is a prospective registry conducted by the Jagiellonian University Medical College in Krakow in collaboration with the Association of Cardiovascular Interventions of the Polish Cardiac Society. Although participation in the ORPKI database is optional, nearly all catheterization laboratories in Poland (98%) submit their data. The detailed description of the registry was presented in previous papers [9,10,11]. Currently, there are more than 160 catheterization laboratories in Poland that are reporting data for the ORPKI registry online. For this analysis, data on all percutaneous procedures (angiography or PCI) were extracted for an 8-year period. The study cohort comprised all adults undergoing angiography and/or PCI between 2014 and 2022 derived from the ORPKI registry and divided into groups with FFR-guided and angio-guided procedure and stratified by sex. We have retrospectively analyzed the data of more than 1.4 million angiography and/or PCI procedures [1,454,121 patients (62.54% men and 37.46% women)]. We evaluated the baseline characteristics, procedural outcomes, and the prevalence of peri-procedural complications. Baseline characteristics and outcomes were reported for patients with and without FFR-guided procedures (coronary angiography (CAG) and CAG followed by PCI). Only peri-procedural outcomes and complications were recorded in the database. The utilization of FFR, as well as all the technical aspects of the procedure, e.g., vascular access and treatment strategy, were left to the discretion of the operator. Pharmacological therapy during and after the procedure was prescribed at operator’s discretion. The Bioethics Committee’s approval was not required because of the retrospective nature of the study and anonymization of the collected data. The study flowchart is presented in Figure 1.

### Statistical Analysis

Continuous variables were presented as mean with standard deviation (SD) or median with the first and the thigh quartile (Q1–Q3) for normally and non-normally distributed variables and were compared using Student’s *t*-test or Mann–Whitney U test, respectively. Categorical variables were presented as counts and percentages and compared with the chi-square test. Logistic regression analysis was performed to explore whether female sex was associated with FFR utilization. Firstly, all demographics, baseline, and procedural characteristics were included in univariable models, then variables of clinical importance or with *p*-value less than 0.2 were included for multivariable analysis. This threshold was chosen to ensure that only variables with a statistically significant association with the outcome (FFR utilization) were included in the multivariable model. The multivariable logistic regression model was adjusted for potential confounders identified in the univariate analysis and based on clinical relevance. These variables were chosen because they are known to influence FFR utilization and could potentially confound the relationship between sex and FFR use. The final model was obtained through the minimization of Akkaike Information Criterion (AIC). Multicolinearity was assessed using variance inflation factors (VIF). Model validation was performed using bootstrap resampling. Statistical analysis was performed in R (R Foundation for Statistical Computing, Austria, Vienna, 2023) version 4.3.1 with package ‘rms’ version 6-7.0.

## 3. Results

Baseline, procedural characteristics, and outcomes of patients undergoing FFR-guided procedures compared with individuals undergoing procedures without FFR have been described in Table S1.

**Figure 2:**
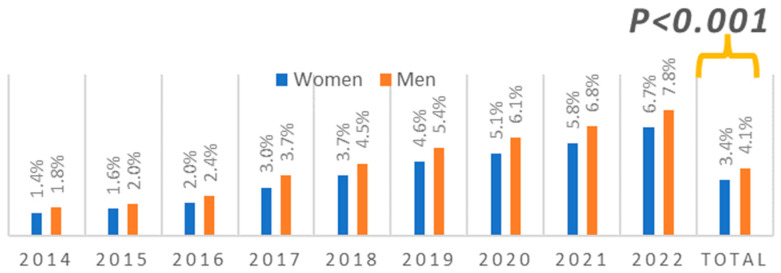
Temporal changes and differences in the number of FFR-guided procedures between men and women in years 2014–2022.

The FFR-guided procedure was performed in 61,305 (4.22%) patients and more frequently in men than women (4.15% vs. 3.45%, respectively, *p* < 0.001). FFR was less often performed in acute coronary syndrome (ASC) than in chronic coronary syndrome (CCS) (24.85% vs. 75.15%, *p* < 0.001). Differences in the number of FFR-guided procedures between men and women in the years 2014–2022 have been illustrated in Figure 2.

Females with FFR-guided procedures were older than men [69.07 (±8.87) vs. 65.45 (±9.38) *p* < 0.001], and more frequently diabetic (24.07% vs. 20.45%, *p* < 0.005); however, they less often had a history of myocardial infarction (MI) (24.79% vs. 36.73%, *p* < 0.001), CABG (1.62% vs. 2.55%, *p* < 0.005), or PCI (36.6% vs. 24.79%, *p* < 0.001) compared to men undergoing FFR. The disparities between men and women who underwent FFR-guided procedures have been shown in Table 1. Crude comparison has shown that male sex was associated with a higher frequency of FFR assessment (OR = 1.2152, 1.1945–1.2361, *p* < 0.005). Factors associated with performing FFR, the results of univariate analysis, have been shown in Table 2.

Female patients in whom FFR was performed more often had previous MI (24.49% vs. 16.22%, *p* < 0.005), PCI (35.71% vs. 20.20%, *p* < 0.005), and hypertension (74.99% vs. 71.66%, *p* < 0.005) than females who underwent angiography and/or PCI without FFR guidance. Women with FFR-guided procedures were slightly younger when compared to women without FFR assessment (69.22 (±8.92) vs. 69.43 (±10.25), *p* < 0.005). Similarly, men who underwent FFR-guided procedure were older (65.54 (±9.40) vs. 65.26 (±10.85), *p* < 0.005), less often smokers (18.97% vs. 20.84%, *p* < 0.005), more frequently with a history of myocardial infarction (MI) (35.8% vs. 24.84%, *p* < 0.005), hypertension (67.73% vs. 71.65%, *p* < 0.005), and previous PCI when compared to male patients that did not underwent FFR.

Female and male patients alike had FFR performed less frequently in ACS setting. In group of patients with ACS, FFR was more frequently assessed in females than males (27.75% vs. 26.08%, *p* < 0.001). Patients with multivessel disease (MVD) or without any significant stenosis less often underwent FFR-guided procedures.

**Figure 3:**
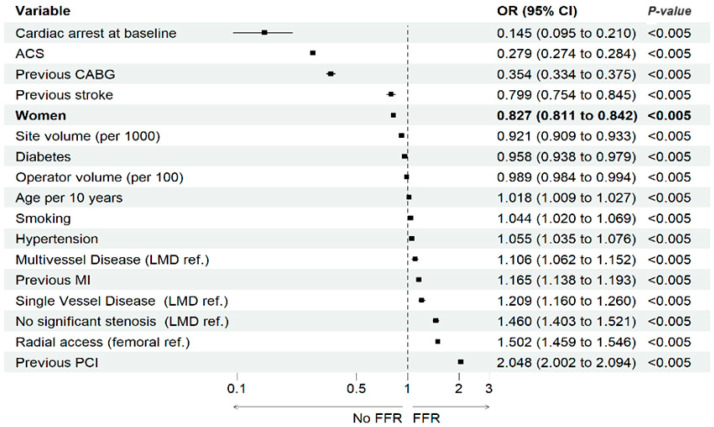
Independent factors associated with FFR utilization–multivariable analysis.

In the multivariable analysis, adjusted for baseline, clinical, procedural, and angiographic characteristics, as well as operator/site volume, female sex was associated with a lower probability of performing FFR (odds ratio of 0.827; 95% confidence interval 0.811–0.842, *p* < 0.001). Other independent factors associated with FFR utilization have been shown in Figure 3.

The multivariable analysis identified several independent predictors of FFR utilization. Notably, patients presenting with acute coronary syndrome (ACS) (OR: 0.279, 95% CI: 0.274 to 0.284, *p* < 0.005) and those experiencing cardiac arrest at baseline (OR: 0.145, 95% CI: 0.095 to 0.210, *p* < 0.005) were less likely to have FFR performed. Conversely, factors such as previous percutaneous coronary intervention (PCI) (OR: 2.048, 95% CI: 2.002 to 2.094, *p* < 0.005) and noncritical stenosis (OR: 1.460, 95% CI: 1.403 to 1.521, *p* < 0.005) were positively associated with FFR utilization. Patients with a history of previous coronary artery bypass grafting (CABG) (OR: 0.354, 95% CI: 0.334 to 0.375, *p* < 0.005) and previous stroke (OR: 0.799, 95% CI: 0.754 to 0.845, *p* < 0.005) are less likely to undergo FFR. Additionally, factors such as multivessel disease (OR: 1.106, 95% CI: 1.062 to 1.152, *p* < 0.005) or previous myocardial infarction (MI) (OR: 1.165, 95% CI: 1.138 to 1.193, *p* < 0.005) are associated with higher odds of FFR utilization.

## 4. Discussion

In the present large-scale, retrospective analysis of data derived from an extensive Polish registry of percutaneous coronary interventions, we have evaluated sex-related disparities in FFR utilization in patients who underwent angiography and/or PCI. The key finding of our study is that despite a substantial rise in FFR utilization, adoption in women is still lower than in men. Female sex was found to be an independent negative predictor of FFR use.

The reason for the underrepresentation of women in CAD trials is unclear. Nonetheless, analysis of large clinical trials in which enrollment of women did not exceed 27% (ISCHEMIA, PROMISE, COURAGE, ORBITA, FAME, and FAME 2 trial) gives a better understanding of the factors that contribute to this disproportion, including sex disparities in obstructive CAD prevalence, clinical presentations, and outcomes, as well as inadequate representation of women in leadership positions [12,13,14,15,16,17,18,19,20].

Atherosclerotic plaques in women have a lower tendency to rupture, smaller lumen area, reduced necrotic core burden, and more diffuse pattern than in male patients [21]. Furthermore, the frequency of spontaneous coronary artery dissection is higher in women [22]. Female patients are also more prone to impaired coronary flow reserve and microvascular dysfunction, which are associated with poorer cardiovascular outcomes [23]. Numerous studies have reported that females less often have complex coronary artery disease than men. In the SYNTAX trial, women constituted only 22.3% of patients and had lower SYNTAX score (mean 29.2 ± 11.1 vs. 27.0 ± 12.2; *p* = 0.001), lower rates of total occlusion (24.7% vs. 18.1%; *p* = 0.006 bifurcation lesions (74.4% vs. 66.8%), and shorter total stent length in the non-surgical arm when compared to men. It is important to emphasize that older age and comorbidities contribute to the lower odds of females being referred for coronary angiography than men. Therefore, the data are likely to underestimate the true frequency of complex coronary artery disease in women [13].

“Yentl syndrome”, first found by Dr B. Healy more than three decades ago, describes sex-related healthcare differences, especially in the field of cardiology, including underdiagnosis or delay in diagnosis as well as undertreatment in female patients. It prompted a significant shift in the approach to cardiovascular health in women, increasing awareness and broadening research on cardiovascular sex-related disparities [21,24]. The Women’s Ischemia Syndrome Evaluation (WISE) study, designed to explore the etiology of myocardial ischemia in the absence of epicardial coronary artery stenoses in women, showed that females with suspected ischemia undergoing coronary angiography with obstructive CAD had a higher prevalence of atypical angina, which was associated with higher long-term mortality [25,26]. Despite women presenting with a lower atherosclerotic disease burden and lesion complexity, they remain at high risk of major adverse cardiac events after PCI, making evidence-based treatments in this population essential [13]. Given that CAD in women has a unique phenotype, including higher resting flow, higher prevalence of nonobstructive coronary artery disease and less calcified lesions, the absence of sex-specific guidelines for treating CAD in women is problematic, especially in CCS setting.

Several studies indicated that women with CAD are often underdiagnosed or suffer from a substantial delay in diagnosis, which might lead to the undertreatment of female patients and have far-reaching consequences for their outcomes [27]. FFR guidance is proven to be a safe and efficient method to assess the significance of a lesion and to improve outcomes, yet the global utilization rate of physiologic assessment with FFR is less than 6%. Thus, it is crucial to increase the number of FFR-guided procedures for both men and women. Even though the present analysis confirmed the annual surge in FFR utilization in Polish patients, taking into consideration the benefits as well as cost-effectiveness of FFR shown in numerous studies, action should be taken to further increase the number of FFR-guided procedures and to improve the long-term prognosis for both men and women [28].

A higher prevalence of microvascular dysfunction in females justifies greater FFR values in comparison with men with similar severity of stenoses. For that reason, applying a rigid FFR cut-off value for all patients might not be beneficial; however, further analysis is necessary [29]. The current analysis has shown that women who underwent FFR-guided procedures were more often diabetic. Studies have indicated that patients with diabetes are at high risk, even with normal FFR value. FFR evaluation might underestimate the risk because of the diffuse coronary artery disease or microvascular dysfunction in the diabetic population, which decreases the hyperemia that can be obtained with adenosine. Thus, further studies are needed to assess whether FFR thresholds for women should change [30].

The utilization of FFR in acute vs. chronic coronary syndrome settings also shows notable disparities. These differences can be attributed to various factors including clinical presentation, physician bias, and adherence to guidelines. Patients with ACS, such as those experiencing myocardial infarction, often present with urgent, life-threatening symptoms that necessitate immediate revascularization. In these cases, there is limited time to perform FFR due to the need for rapid intervention to restore blood flow and minimize myocardial damage. In contrast, CCS patients typically have stable symptoms like angina, allowing for a more comprehensive diagnostic approach. This setting is more conducive to performing FFR to guide the decision-making process for potential revascularization.

Physicians may perceive the risks of delaying revascularization for FFR measurement in ACS patients as outweighing the benefits. The immediate need to address the acute event can lead to a bias against performing additional diagnostic procedures like FFR. In a stable setting, the potential benefits of FFR, such as avoiding unnecessary revascularizations and optimizing medical therapy, are more apparent, leading to higher utilization rates.

Some reports indicate that clinicians may have a higher threshold for initiating the diagnostic workup and treatment for suspected CCS in females, with 25% of women being asymptomatic for chest pain at the time of referral compared to 33% of men [31].

Variability in institutional protocols and the availability of FFR technology can also influence utilization rates. Physicians’ familiarity with FFR may vary. Hospitals with established protocols that incorporate FFR in the management of CCS are more likely to adhere to guidelines and use FFR regularly compared to those that focus primarily on ACS interventions.

A significant factor contributing to the underutilization of FFR could be delays in diagnosis, likely due to symptom misrecognition or women not seeking immediate help from emergency services. This can lead to more severe or acute conditions where FFR is less indicated. What is more, insufficient operator expertise in instances of non-obstructive coronary artery disease may result in FFR not being utilized, which could lead to misdiagnosis or incomplete evaluation of microcirculation.

The multivariable analysis underscores important disparities and clinical factors influencing FFR utilization. Our findings demonstrate that female sex is a significant independent predictor of lower FFR use, aligning with previous studies reporting sex-related differences in cardiovascular diagnostic procedures. Additionally, patients presenting with ACS and those with a history of cardiac arrest at baseline are less likely to undergo FFR, suggesting that the urgency and severity of their condition might deter the use of this diagnostic tool. Conversely, patients with previous PCI and those with noncritical stenosis were more likely to have FFR performed, reflecting clinical practice trends where FFR is utilized to guide treatment strategies in less acute settings. These findings highlight the need for targeted strategies to address these disparities and optimize FFR use across different patient populations.

The improving trends in FFR utilization seen in our study are reassuring, but further initiatives are needed nationwide to eliminate sex-related disparities. Spreading awareness and implementing nationwide quality improvement initiatives are necessary to reduce these disparities further. The reasoning behind the sex-related discrepancies is complex. Numerous studies have shown that male and female patients differ in terms of lesion morphology and symptomatology, which might influence the operator’s decision-making process.

### Limitations

The findings of the current analysis must be interpreted in light of some limitations. Firstly, the ORPKI database is a prospective registry, and its observational nature is a restriction that arises from the study design. Due to the observational character of the study, we cannot exclude a residual confounder. Despite being a large sample representative of most of the Polish population, the participation in the ORPKI database is optional and submitting the data relies on the operator. In our study, the FFR group includes patients who underwent FFR as part of their diagnostic and treatment process, regardless of whether it was performed in isolation or as part of a comprehensive physiological assessment. Our dataset does not differentiate between these scenarios, which might be a potential source of bias. Finally, only periprocedural and not long-term data was available in the ORPKI database. Despite limitations, the current analysis has several strengths. To our knowledge, this is the first study that evaluates sex-based differences in the utilization of FFR in patients undergoing coronary angiography and PCI.

## 5. Conclusions

This large-scale registry-based analysis has identified significant sex-based disparities in FFR utilization. Women are significantly less likely to undergo FFR evaluation than men. Despite a substantial rise in FFR use throughout years, adoption in women remains low, resulting in fewer FFR-guided revascularizations for women, contrary to current ESC recommendations.

## Figures and Tables

**Table 1:** Comparison of populations of men and women who underwent FFR-guided procedures.

	Female, *n* = 18,860	Male, *n* = 37,647	*p* Value
Age, years	69.07 (±8.87)	65.45 (±9.38)	<0.005
Diabetes, %	24.07	20.45	<0.005
Previous stroke, %	2.06	2.39	0.011
Previous MI, %	24.79	36.73	<0.005
Previous PCI, %	36.60	48.20	<0.005
Previous CABG, %	1.62	2.55	<0.005
Smoking status—smoker, %	12.15	18.88	<0.005
Hypertension,%	75.38	72.12	<0.005
Kidney disease, %	4.85	4.67	0.352
COPD,%	2.47	2.89	<0.005
ACS, %	27.75	26.08	<0.005
Access site during angiogram			
Femoral,%	11.96	8.93	<0.005
Radial,%	88.04	91.07	<0.005
Results of angiography			<0.005
LMCA%	3.17	6.18	
MVD%	19.77	27.85	
SVD,%	23.40	25.78	
No significant stenosis,%	53.66	40.18	
Cardiac arrest at baseline, %	0.04	0.05	0.563
Operator volume annual, Me (Q1–Q3)	296.89 (196.89; 421.67)	291.44 (193.78; 421.00)	0.033
Site volume annual, Me (Q1–Q3)	1293.44 (1015.44; 1621.56)	1293.44 (1015.44; 1635.33)	0.048
Periprocedural stroke, %	0.00	0.02	0.011
Dissection, %	0.08	0.07	0.597
Bleeding at the puncture site, %	0.03	0.02	0.461
Cardiac arrest during procedure, %	0.11	0.06	0.097
Allergic reaction during procedure, %	0.02	0.02	0.819
Death during procedure, %	0.02	0.02	0.998

Abbreviations: ACS, acute coronary syndrome; CABG, coronary artery bypass grafting; COPD, chronic obstructive pulmonary disease; LMCA, left main coronary artery; Me, median; MI, myocardial infarction; MVD, multivessel disease; PCI, percutaneous coronary intervention.

**Table 2:** Univariable analysis. Factors associated with use of FFR.

	OR	Lower	Upper	*p* Value
Sex (male)	1.22	1.19	1.24	<0.005
Age, years	0.10	0.10	1.00	0.119
Diabetes	0.10	0.98	1.02	0.950
Previous stroke	0.78	0.74	0.83	<0.005
Previous MI	1.71	1.68	1.74	<0.005
Previous PCI	2.20	2.17	2.24	<0.005
Previous CABG	0.39	0.37	0.41	<0.005
Hypertension	1.19	1.17	1.21	<0.005
Kidney disease	0.96	0.92	0.99	0.014
COPD	1.10	1.05	1.16	<0.005
Smoking status (smoker)	0.95	0.93	0.97	<0.005
Indications (CCS ref.)				
UA	0.39	0.38	0.39	<0.005
ACS	0.27	0.27	0.28	<0.005
NSTEMI	0.14	0.14	0.15	<0.005
STEMI	0.02	0.02	0.03	<0.005
Chronic heart failure	0.89	0.85	0.94	<0.005
Acute heart failure	0.48	0.41	0.56	<0.005
Cardiac arrest treatment	0.12	0.10	0.15	<0.005
Results of coronary angiography				
Only LMCA	0.67	0.58	0.77	<0.005
Single Vessel Disease	0.61	0.60	0.62	<0.005
Without significant stenosis	0.12	0.11	0.13	<0.005
Multi Vessel Disease	0.52	0.51	0.53	<0.005
Multi Vessel Disease with LMCA	0.41	0.40	0.43	<0.005
Direct transport	0.06	0.05	0.07	<0.005

Abbreviations: ACS, acute coronary syndrome; CABG, coronary artery bypass grafting; CCS, chronic coronary syndrome; COPD, chronic obstructive pulmonary disease; LMCA, left main coronary artery; MI, myocardial infarction; NSTEMI, non-ST-elevation myocardial infarction; PCI, percutaneous coronary intervention; STEMI, ST-elevation myocardial infarction; UA, unstable angina; ref., reference.

## Data Availability

The raw data supporting the conclusions of this article will be made available by the authors on request.

## Supplementary Materials

The following supporting information can be downloaded at: https://www.mdpi.com/article/10.3390/jcm13144028/s1, Table S1. Baseline, Procedural Characteristics, and Outcomes of Patients Undergoing FFR guided procedures compared with individuals undergoing procedures without FFR.
