# Supplementary material for: Sex Differences in Fractional Flow Reserve Utilization
Source: J Clin Med. 2024 Jul 10;13(14):4028. doi: 10.3390/jcm13144028 (PMC11277883; doi:10.3390/jcm13144028)
Supplement: Supplementary file 1 [file jcm-13-04028-s001.zip › jcm-3036066-supplementary.pdf]

Journal of Clinical Medicine

MDPI

Supplementary Material

Table S1. Baseline, Procedural Characteristics, and Outcomes of Patients Undergoing FFR guided procedures compared with individuals undergoing procedures without FFR.

	Men: No FFR	Men: FFR	p value	Women: No FFR	Women: FFR	p value
Number of patients	957819 (95.90%)	40962 (4.10%)		578030 (96.60%)	20343 (3.4%)	
Age, years	65.26 (±10.85)	65.54 (±9.40)	<0.005	69.43 (±10.25)	69.22 (±8.92)	<0.005
Diabetes,%	20.53	20.52	0.9651	23.60%	24.04	0.151
Previous stroke,%	3.05	2.46	<0.005	2.86	2.10	<0.005
Previous MI, %	24.84	35.80	<0.005	16.22	24.49	<0.005
Previous PCI,%	28.76	46.68	<0.005	20.20	35.71	<0.005
Previous CABG, %	6.84	2.56	<0.005	3.56	1.61	<0.005
Smoking status – smoker,%	20.84	18.97	<0.005	11.67	12.09	0.065
Hypertension,%	67.73	71.65	<0.005	71.66	74.99	<0.005
Kidney disease,%	5.23	4.99	0.0295	5.45	5.25	0.222
COPD,%	2.82	3.00	0.0345	2.22	2.57	<0.005
Indication for the procedure						<0.005
Stable angina,%	36.19	68.69	<0.005	39.00	67.86	
UA,%	28.76	20.09	<0.005	30.31	22.00	
ACS,%	55.55	24.25	<0.005	53.47	26.07	<0.005
NSTEMI,%	14.00	3.60	<0.005	12.96	3.64	
STEMI,%,	12.78	0.55	<0.005	10.19	0.43	
Chronic heart failure,%	2.09	3.29	<0.005	1.24	2.10	
Acute heart failure,%	0.35	0.31	<0.005	0.26	0.21	
Cardiac arrest ,%	0.94	0.21	<0.005	0.64	0.13	
Thrombolysis during angiogram,%	0.01	0.00	0.0238	0.01	0.00	0.725
Total amount of contrast used during procedure, ccm, (±SD)	127.01 (±78.18)	128.35 (±73.05)	<0.005	106.57 (±69.79)	114.86 (±63.95)	<0.005
Total radiation dose during procedure, mGy, (±SD)	710.31 (±776.77)	647.60 (±624.55)	<0.005	488.22 (±622.29)	461.95 (±491.60)	<0.005
Access site during angiogram						
Femoral,%	15.95	8.82	<0.005	17.82	11.84	<0.005
Radial,%	83.04	90.05	<0.005	81.00	86.81	<0.005
		Results of angiography				
Multi Vessel Disease,%	33.65	27.75	<0.005	24.42	19.88	<0.005
Multi Vessel Disease with LMCA,%	8.82	5.87	<0.005	4.98	2.94	<0.005
No obstructive CAD,%	23.04	38.64	<0.005	32.66	51.28	<0.005
Only LMCA,%	0.31	0.34	<0.005	0.23	0.23	<0.005
Single Vessel Disease,%	26.69	25.72	<0.005	23.29	23.31	<0.005
Without significant stenosis,%	7.49	1.68	<0.005	14.43	2.37	<0.005
Operator volume total, mean (±SD)	2900.79 (±1634.3)	2904.39 (±1605.7)	0.6620	2922.57 (±1635.69)	2935.31 (±1621.6)	0.275

**Site volume total, mean (±SD)**	**12688.53 (±6185.0)**	**12525.61 (±5609.4)**	**<0.005**	**12591.24 (±6145.81)**	**12462.96 (±5536.4)**	**<0.005**
**Periprocedural Stroke,%**	**0.01**	**0.02**	**0.3981**	**0.02**	**0.00**	**0.052**
**Dissection,%**	**0.06**	**0.07**	**0.1688**	**0.09**	**0.09**	**1.000**
**Bleeding at the puncture site %**	**0.02**	**0.03**	**0.3917**	**0.05**	**0.04**	**0.443**
**Cardiac arrest during procedure,%**	**0.19**	**0.06**	**<0.005**	**0.19**	**0.10**	**<0.005**
**Death during procedure,%**	**0.25**	**0.01**	**<0.005**	**0.29**	**0.01**	**<0.005**
**Any complication,%**	**1.07**	**0.44**	**<0.005**	**1.22**	**0.52**	**<0.005**

Abbreviations: ACS, acute coronary syndrome; CABG, coronary artery bypass grafting; COPD, chronic obstructive pulmonary disease; LMCA, left main coronary artery; LVEF, left ventricular ejection fraction; MI, myocardial infarction; MVD, Multivessel disease; NSTEMI, non-ST-elevation myocardial infarction; PCI, percutaneous coronary intervention; RCA, right coronary artery; SD, standard deviation; STEMI, ST-elevation myocardial infarction; UA, unstable angina;
